# Murine neuronatin deficiency is associated with a hypervariable food intake and bimodal obesity

**DOI:** 10.1038/s41598-021-96278-8

**Published:** 2021-09-02

**Authors:** Irene Cimino, Debra Rimmington, Y. C. Loraine Tung, Katherine Lawler, Pierre Larraufie, Richard G. Kay, Samuel Virtue, Brian Y. H. Lam, Luca Fagnocchi, Marcella K. L. Ma, Vladimir Saudek, Ilona Zvetkova, Antonio Vidal-Puig, Giles S. H. Yeo, I. Sadaf Farooqi, J. Andrew Pospisilik, Fiona M. Gribble, Frank Reimann, Stephen O’Rahilly, Anthony P. Coll

**Affiliations:** 1 MRC Metabolic Diseases Unit, University of Cambridge Metabolic Research Laboratories, Wellcome Trust-MRC Institute of Metabolic Science, Addenbrooke’s Hospital, Cambridge, CB2 0SL UK; 2 University of Cambridge Metabolic Research Laboratories and NIHR Cambridge Biomedical Research Centre, Wellcome Trust‑MRC Institute of Metabolic Science, Addenbrooke’s Hospital, Cambridge, CB2 0SL UK; 3 Université Paris-Saclay, AgroParisTech, INRAE, UMR PNCA, 75005, Paris, France; 4 Department of Epigenetics, Van Andel Institute, Grand Rapids, MI 49503 USA

**Keywords:** Genetics, Molecular biology, Neuroscience, Physiology

## Abstract

Neuronatin (*Nnat*) has previously been reported to be part of a network of imprinted genes downstream of the chromatin regulator Trim28. Disruption of Trim28 or of members of this network, including neuronatin, results in an unusual phenotype of a bimodal body weight. To better characterise this variability, we examined the key contributors to energy balance in *Nnat*^+*/−p*^ mice that carry a paternal null allele and do not express *Nnat*. Consistent with our previous studies, *Nnat* deficient mice on chow diet displayed a bimodal body weight phenotype with more than 30% of *Nnat*^+*/−p*^ mice developing obesity. In response to both a 45% high fat diet and exposure to thermoneutrality (30 °C) *Nnat* deficient mice maintained the hypervariable body weight phenotype. Within a calorimetry system, food intake in *Nnat*^+*/−p*^ mice was hypervariable, with some mice consuming more than twice the intake seen in wild type littermates. A hyperphagic response was also seen in *Nnat*^+*/−p*^ mice in a second, non-home cage environment. An expected correlation between body weight and energy expenditure was seen, but corrections for the effects of positive energy balance and body weight greatly diminished the effect of neuronatin deficiency on energy expenditure. Male and female *Nnat*^+*/−p*^ mice displayed subtle distinctions in the degree of variance body weight phenotype and food intake and further sexual dimorphism was reflected in different patterns of hypothalamic gene expression in *Nnat*^+*/−p*^ mice. Loss of the imprinted gene *Nnat* is associated with a highly variable food intake, with the impact of this phenotype varying between genetically identical individuals.

## Introduction

Genomic imprinting is the mechanism by which a gene is expressed only by one parental allele, while the other allele is silenced through epigenetic modification^1^. Imprinted genes have an essential role in embryonic development and postnatal growth and are increasingly recognised to influence metabolism beyond early life, with involvement in energy homeostasis and body weight composition into adulthood (reviewed by^2,3^).There is also strong evidence that imprinted genes have a role in higher cognitive processes, reward-based processing and behavioural phenotypes^4^.

Neuronatin (*Nnat*) is an imprinted gene transcribed exclusively from the paternal allele. It is located in a micro-imprinted genomic locus situated within the first intron of another imprinted gene, *Blcap* (Bladder Cancer Associated Protein)^5^.

*Nnat* was first described in studies of the developing brain^6,7^. In adult hypothalamic nuclei expression is influenced by metabolic status^8^ and recognised to be downregulated in obese *ob/ob* and *db/db* mice^9,10^. Subsequent reports have shown expression in several peripheral sites including pituitary, pancreas and adipocytes^11–13^, although the physiological and cellular role of *Nnat* at these sites remains to be fully determined. Nnat has been characterised and reported to be an amphiphilic membrane proteolipid highly homologous to mammalian sarcoplasmic protein Phospholamban (Pln) and yeast protein PM1^14–16^. However, this model was based on a sequence alignment where the Nnat amino acid sequence was written backwards and, in an effort to obtain maximal similarity, a number of gaps were introduced^17^.

We have reported *Nnat* to be one of a subset of paternal expressed genes downregulated in obese mice haploinsufficient for *Trim28*, the gene encoding the chromatin-interacting protein TRIM28^18^. Haploinsufficient *Trim28* mice display a bimodal body weight phenotype, emerging into adulthood as either obese or normal body weight. Further, we have described that mice inheriting a null *Nnat* allele from their father (*Nnat*^+*/−p*^) also exhibit a bimodal body weight, with around a quarter of adult *Nnat*^+/−p^ mice having an increase in body weight characterised by a higher fat mass^18^. A more recent study using an independent C57BL/6J mouse line deficient in *Nnat* did not report a body weight phenotype but did describe how loss of *Nnat* impaired glucose-stimulated insulin secretion, with a putative role for neuronatin in facilitating processing of pre-proinsulin^19^. However, in a follow up study of mice carrying the same mutant allele, adult *Nnat*^+/−p^ mice did display a bimodal body weight phenotype, albeit one with a subpopulation of lean, as opposed to obese, mice^20^. Intriguingly, when the mutant allele was studied in a 129S2/Sv background, mice deficient in *Nnat* displayed both postnatal growth restriction with diminished adiposity and an obesity phenotype in adulthood.

These results indicate that although a loss of *Nnat* can impact upon body weight, a full understanding of the physiological function of neuronatin still remains elusive. To further investigate the role of *Nnat* in feeding behaviour and body composition we have undertaken phenotypic characterisation of *Nnat* null animals rederived into a second, independent centre distinct from the site in which our original studies were undertaken. We also performed transcriptomic analysis of hypothalamic tissue and peptidomic analysis of pancreatic tissue from *Nnat* null animals.

## Results

### *Nnat* deficient mice have a highly variable body weight phenotype that persists on a high fat diet and at thermoneutrality

**Figure 1:**
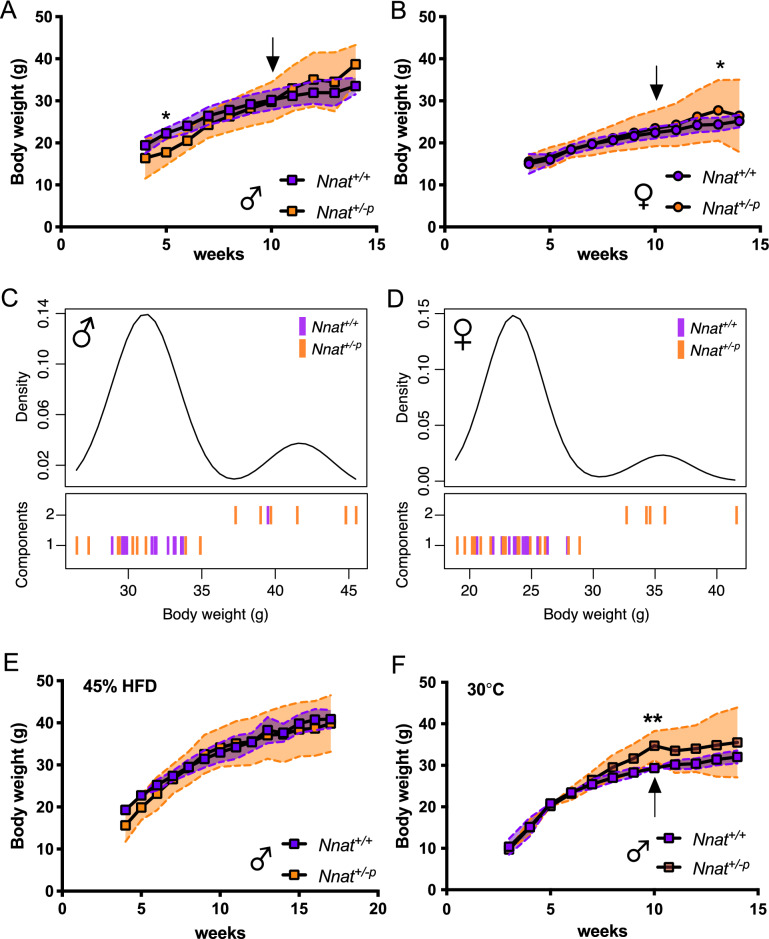
*Nnat* deficient mice have a highly variable body weight phenotype. Body weight over time of *Nnat*^+*/*+^ and *Nnat*^+*/−p*^ (**A**) male and (**B**) female mice on standard chow diet (at least 7 mice were included in each time point per genotype). Black arrow indicating time of the initiation of single housing. Mclust clustering showed significant variance of body weight at 12 weeks of age between *Nnat*^+*/*+^ and *Nnat*^+*/−p*^ male (**C**) and females (**D**) mice (male; *Nnat*^+*/*+^ (n = 19), Nnat^+*/−p*^ (n = 17): females; *Nnat*^+*/*+^ (n = 16), *Nnat*^+*/−p*^ (n = 21)). (**E**) Body weight over time of *Nnat*^+*/*+^ and *Nnat*^+*/−p*^ male mice fed 45% HFD (at least 7 mice at each time point per genotype). (**F**) Body weight over time of *Nnat*^+*/*+^ and *Nnat*^+*/−p*^ male mice at thermoneutral condition (30 °C) fed on standard chow diet (at least 7 mice at each time point per genotype). Black arrow indicating time of the initiation of single housing. Data are expressed as mean (square-circle points) ± SD (dotted lines), *P < 0.05; **P < 0.01, 2-way ANOVA with multiple comparison.

Previous reports have shown global loss of *Nnat* affects both early growth and final adult body weight^18,20,21^. We measured the body weight of *Nnat*^+*/−p*^ and *Nnat*^+*/*+^ mice from weaning and found that during the early postnatal period (4–6 weeks), male *Nnat*^+*/−p*^ mice had both a significant reduction in mean body weight and a significant increase in body weight variance (Fig. 1A, Supplementary Fig. 1A). Interestingly, in female mice at this time point there were no significant differences in body weight mean or variance (Fig. 1B, Supplementary Fig. 1B). By around 9 weeks old, *Nnat*^+*/−p*^ mice matched the mean body weight of their *Nnat*^+*/*+^ littermates. By 12 weeks of age, there was a significant increase in the variance of body weight in *Nnat*^+*/−p*^ mice compared to *Nnat*^+*/*+^ mice (Supplementary Fig. 1C,D). 35% of *Nnat*^*+/−p*^ males and 33% of *Nnat*^+*/−p*^ females developed obesity (defined as greater than 2 standard deviation (SD) of *Nnat*^+*/*+^ mean) (Supplementary Fig. 1C, D), while the remaining mice displayed a body weight similar to *Nnat*^+*/*+^.

We also applied an unbiased model-based clustering approach^22^ to the body weight data of 12 weeks old mice to assess whether they were best fitted by either a single Gaussian or a mixture of normal distribution. This analysis indicated that the body weight data of mice were best modelled by bimodal distributions, in both males and females. Importantly, the *Nnat*^+*/−p*^ animals were highly enriched in the cluster comprising obese mice (Fig. 1C,D).

Previous reports have suggested that variability in body weight seen in *Trim28* haploinsufficient mice may be linked to environmental factors such as ambient temperature and diet. To determine if such factors may influence the magnitude and frequency of obesity in *Nnat*^+*/−p*^ mice, we studied body weight responses of additional, independent cohorts of *Nnat*^+*/−p*^ male mice under two different conditions: 45% high fat diet (HFD) and thermoneutrality (30 °C).

In the HFD experiment, mice were switched at 4 weeks of age from a standard chow diet to 45% HFD and kept on this regimen for 3 months with body weight measured weekly (Fig. 1E). Again, in this male cohort, in the early post-natal period *Nnat*^+*/−p*^ mice showed reduced body weight and increased variance compared to wild type (Supplementary Fig. 2A, B) and at 12 weeks of age, *Nnat*^+*/−p*^ mice displayed a clear increased variance in body weight compared to *Nnat*^+*/*+^ (Supplementary Fig. 2C). Of note, when compared to chow, the overall impact of the 45% HFD on mean body weight at 12 weeks was minimal. Wild type mice had a modest increase in mean body weight (mean ± SEM, chow vs 45%HFD, 31.5 ± 0.4 g vs 34.3 ± 0.7 g) while in *Nnat*^+*/−p*^ mice there was no change (mean ± SEM, chow vs 45% HFD 35.8 ± 1.7 g vs 35.5 ± 2.0 g); these differences were not significant by 2-way ANOVA. We performed again the unbiased model-based clustering approach previously done, but in this case, we could not detect a clear bimodal distribution in *Nnat*^+*/−p*^ mice (Supplementary Fig. 2D). However, when we used a pre-imposed 2 clusters model for the analysis, we could still see an enrichment of *Nnat*^+*/−p*^ mice among the heaviest mice.

In the higher ambient temperature experiment, mice fed on standard chow were weaned at 4 weeks of age into a thermoneutrality cabinet (30 °C) and followed for 3 months (Fig. 1F). No body weight differences were found in postnatal stage (5–6 weeks) between *Nnat*^+*/−p*^ and *Nnat*^+*/*+^ mice (Fig. 1F). The body weight of *Nnat*^+*/−p*^ mice began to differentiate from *Nnat*^+*/*+^ mice at around 8 weeks of age. At 10 weeks old, almost all *Nnat*^+*/−p*^ mice (85%) displayed heavier body weight than *Nnat*^+*/*+^ (Fig. 1F, Supplementary Fig. 2E). Although the cohort size in this study was small, the variance in body weight *Nnat*^+*/−p*^ mice was larger than in *Nnat*^+*/*+^ mice (Supplementary Fig. 2E). Indeed, the model-based clustering analysis gave two clear components present both at 10 and 12-weeks of age, and again *Nnat*^+*/−p*^ mice were enriched among the obese group (Supplementary Fig. 2F, G).

We conclude that the body weight hypervariability observed in *Nnat*^+*/−p*^ mice on chow is maintained both on a high fat diet and at thermoneutrality.

### *Nnat* loss causes hypervariable food intake

In order to investigate the causes of the bimodal obesity phenotype in the *Nnat*^+*/−p*^ mice we performed indirect calorimetry experiments (Meta Trace) to simultaneously measure food intake (FI) and energy expenditure (EE).

**Figure 2:**
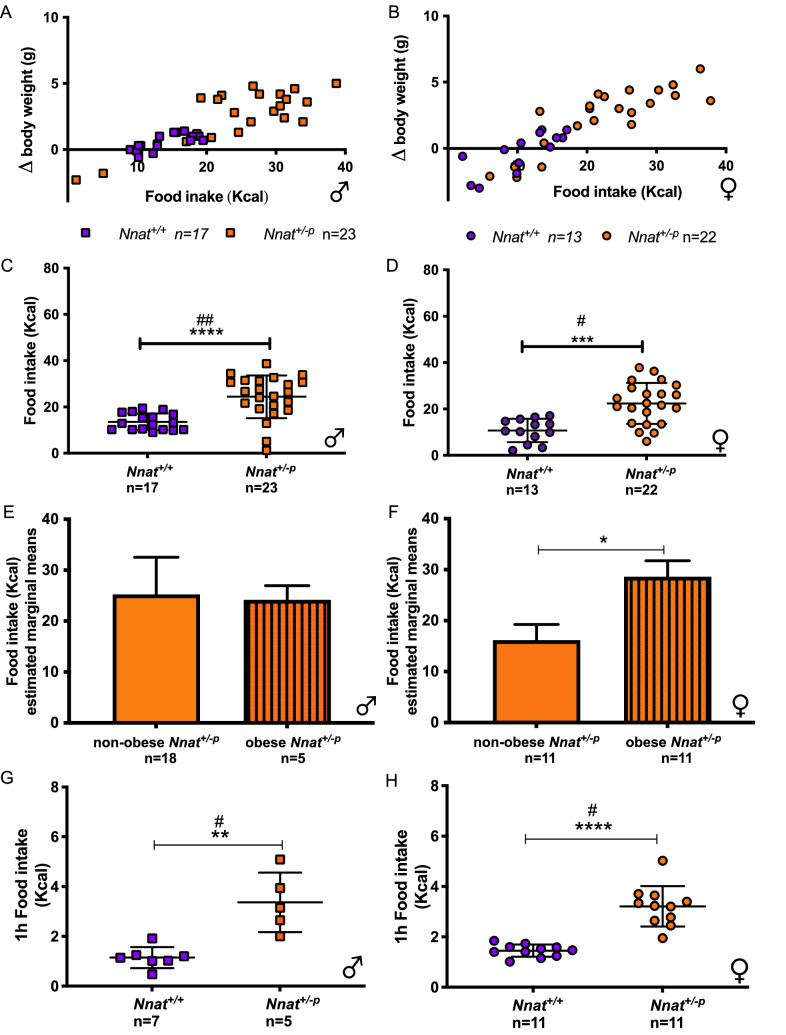
*Nnat* loss affects food intake. Change in body weight plotted against food intake over 48 h in calorimetry system in male (**A**) and female (**B**) mice fed on standard chow diet. Total food intake in *Nnat*^+*/*+^ and *Nnat*^+*/−p*^ male (**C**) and female (**D**) mice over 48 h in calorimetry system. ANCOVA analysis of the food intake over 48 h in calorimetry system in males (**E**) and females (**F**). Total caloric intake in the re-feeding protocol in male (**G**) and female (**H**) mice fed condensed milk. Data are expressed as mean ± SD, *P < 0.05; **P < 0.01; ***P < 0.001; ****P < 0.0001 for Mann–Whitney test and ^#^*P* < 0.05; ^##^*P* < 0.01 for Levine's test.

To attempt to reduce the impact of the calorimetry environment itself on measured variables we acclimatised mice by singly housing them for 2 weeks (between 10 and 12 weeks of age) in identical cages to those which form the housing component of the calorimetry system. We observed only small changes in body weight in both male and female *Nnat*^+*/*+^ mice during the 48 h calorimetry run (dBW_males_ 0.5 ± 0.6; dBW_females_ − 0.2 ± 1.5 g) (Fig. 2A,B). However, even with this acclimation procedure, over the 48 h period of calorimetry analysis, as a genotype group the male *Nnat*^+*/−p*^ mice gained 2.5 ± 2.0 g (mean ± SEM) while the female mice gained 2.4 ± 2.3 g (Mann–Whitney Test for dBW: *Nnat*^+*/*+^ vs *Nnat*^+*/−p*^: *P* value_males_ = 0.0001; *P* value_females_ = 0.0003) (Fig. 2A,B). Strikingly, during the calorimetric run not only was the mean weight gain greater in *Nnat*^+*/−p*^ mice, but also there was a greater variance in body weight gain in *Nnat*^+*/−p*^ mice than in wild type controls (Levine Test for dBW: *Nnat*^+*/*+^ vs *Nnat*^+*/−p*^: *P *value_males_ = 0.001; *P *value_females_ = 0.17).

There was also a very clear phenotype seen in the food intake of the *Nnat*^+*/−p*^ mice. While male and female wild type mice ate 4.5 ± 0.3 g and 3.2 ± 0.5 g (mean ± SEM, respectively) over the 48 h period, food intake in both male and female *Nnat*^+*/−p*^ mice was hypervariable, with some mice eating more than twice the amount of their wild type littermates, others matching wild type intake and a small number consuming less (Fig. 2C,D). We divided the *Nnat*^+*/−p*^ mice into groups based on their average body weight during calorimetry, making a binary split between those with a body weight 2 standard deviations (SD) greater than the mean of the *Nnat*^+*/*+^ controls labelled “obese”, and those below labelled “non obese”. We found that in female mice, the obese *Nnat*^+*/−p*^ exhibited a greater hyperphagic response in the calorimetry environment than the non-obese *Nnat*^+*/−p*^ (Fig. 2F). In the males this effect was not evident (Fig. 2E), but the distribution of non-obese and obese *Nnat*^+*/−p*^ mice was uneven (18 non obese to 5 obese) whereas the females showed an even 11:11 split. Overall, it appeared that the bimodal weight gain in the calorimeters was both associated with, and showed similar characteristics to, the body weight changes observed in the *Nnat*^+*/−p*^ mice outside of the calorimetry environment, with some differences between males and females.

**Figure 3:**
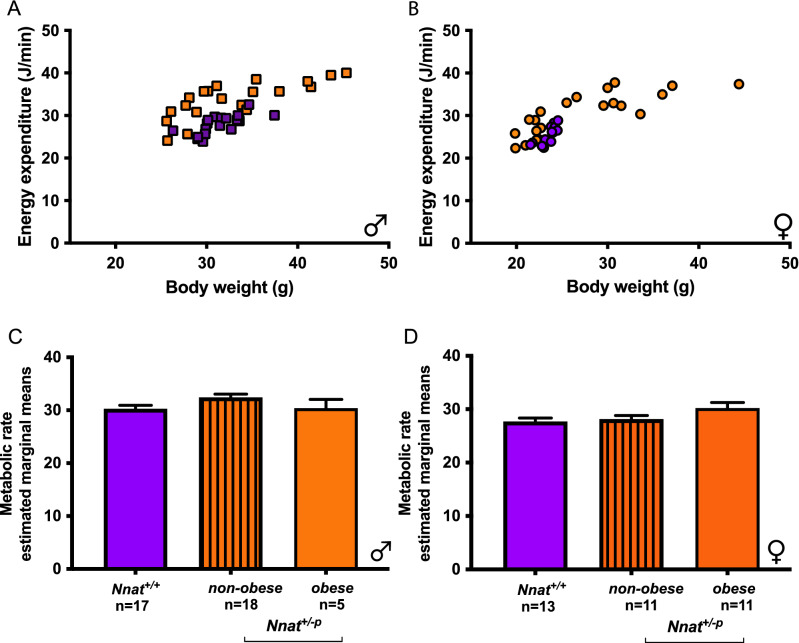
*Nnat* loss affects energy expenditure. Energy expenditure in *Nnat*^+*/*+^ and *Nnat*^+*/−p*^ males (**A**) and female (**B**) mice plotted against body weight (average over time in calorimetry system, mice fed on standard chow diet), wild type blue, *Nnat*^*+/−p *^orange. Comparison of metabolic rate corrected for body weight and dBW using ANCOVA: in male (**C**) and female (**D)** mice. Data are expressed as mean ± SE, *P < 0.01.

Positive energy balance has an energetic cost associated with the thermic effect of feeding. As such, we found strong correlations between food intake, weight gain and energy expenditure across the wild type and *Nnat*^+*/−p*^ mice during the 48 h calorimetry runs. In addition, we found the expected and well-established correlations between body weight and energy expenditure (Fig. 3A,B).

We attempted to correct for the effects of positive energy balance and body weight. To do so we performed an ANCOVA using both body weight (BW) and change in BW (dBW) as covariates. Both dBW and BW were highly significant predictors of EE. Doing so completely eliminated the effects of *Nnat*^+*/−p*^ on energy expenditure in females, with wild types as well as non-obese and obese *Nnat*^+*/−p*^ mice showing the same metabolic rate (Fig. 3D). In the males there was a small increment in metabolic rate in the non-obese *Nnat*^+*/−p*^ mice (Fig. 3C). This increased metabolic rate could explain some of the difference between *Nnat*^+*/−p*^ non-obese and obese groups, however in terms of absolute values, *Nnat*^+*/−p*^ obese mice exhibited 62% more food intake during the calorimetry runs, 27% more energy expenditure and only a 7% lower metabolic rate compared to *Nnat*^+*/−p*^ non-obese mice, suggesting changes in a metabolic rate were only playing a small role in any phenotype (Supplementary Fig. 3A-J). Additionally, it was notable the obese *Nnat*^+*/−p*^ group had an identical metabolic rate to the wild-types.

A small cohort of chow fed mice raised at thermoneutrality were also studied in the calorimetry system at 11–12 weeks of age (Supplementary Fig. 2H,I). Although the pattern of energy expenditure against body weight looked to follow a similar pattern as that seen in mice studied at room temperature, the number of data points was too small to support two covariates or split into “non-obese” or “obese” groups, limiting any further detailed analysis.

Finally, to further study the effects of *Nnat* loss on appetitive behaviour, we examined food intake in a separate cohort in a second experimental paradigm, a ‘non-home cage’ environment to which they had been acclimatised in a fed state beforehand. We fasted *Nnat*^+*/*+^and *Nnat*^+*/−p*^ mice overnight, then introduced individual mice into the Zantiks unit (body weight in Supplementary Fig. 4A,B), where a choice of full fat or light version of condensed milk were freely available. Refeeding consumption was monitored for 1 h. Both male and female *Nnat*^+*/−p*^ mice had a higher total caloric intake (full fat + light, low fat condensed milk Kcal) compared to their *Nnat*^+*/*+^ counterpart controls (Fig. 2G,H). Both male and females *Nnat*^+*/−p*^ mice had a greater intake of the full fat condense milk and light milk compared to *Nnat*^+*/*+^ control mice (Supplementary Fig. 4C,D,E,F).

### *Nnat* loss increases activity levels

In a subset of the chow-fed mice cohort which underwent Meta Trace analysis at room temperature, locomotor activity of the mice was also recorded during the 48 h spent within the calorimetry apparatus. Female *Nnat*^+*/−p*^ mice were more active compared to *Nnat*^+*/*+^ mice (Supplementary Fig. 5B,D), while an increase that did not reach statistical significance was observed in males (Supplementary Fig. 5A,C). The body weight distributions of this subset of analysed mice were comparable with the whole cohort (Supplementary Fig. 5E, F).

### Obese *Nnat* deficient mice have an increase in fat mass

**Figure 4:**
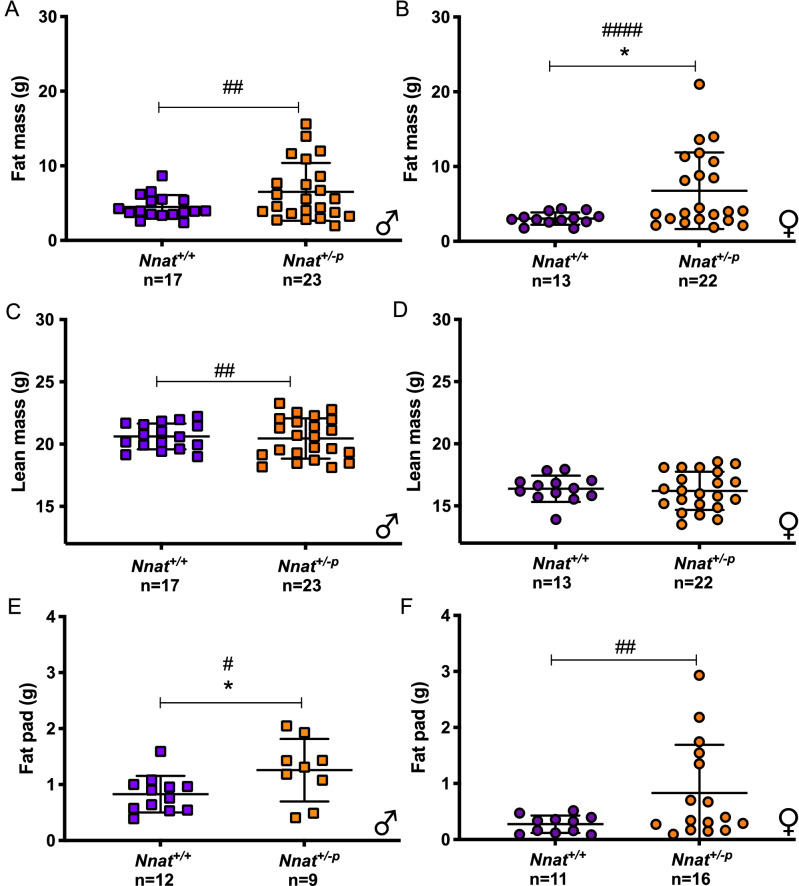
*Nnat* deficient mice display higher fat mass. Body composition analysis of fat and lean mass by TDNMR of male (**A**,**C**) and female (**B**,**D**) *Nnat*^+*/*+^ and *Nnat*^+*/−p*^ mice at 12–13 weeks of age fed on standard chow diet. Weight of inguinal fat pad in male (**E)** and female mice (**F**). Data are expressed as mean ± SD, *P < 0.05 for Mann–Whitney test and ^#^*P* < *0.05*, ^##^*P* < 0.01, ^###^*P* < 0.001 for Levine's test.

The cohort of chow fed mice studied in the Meta Trace subsequently underwent time domain nuclear magnetic resonance (TD-NMR) analysis. In both sexes, there was an increase in mean fat mass in *Nnat*^+*/−p*^ mice compared to their *Nnat*^+*/*+^ counterparts, though it failed to reach statistical significance in the males (*Nnat*^+*/*+^ vs *Nnat*^+*/−p*^: *P* value_*males*_ = 0.14; *P* value_females_ = 0.03) (Fig. 4A,B). As was seen with body weight, the variance of the fat mass distributions was significantly increased in both male and female *Nnat*^+*/−p*^ mice (Fig. 4A,B).

Mean lean mass by TD-NMR was similar between *Nnat*^+*/−p*^ and *Nnat*^+*/*+^ mice for both sexes, but a significant increase and a trend of increase in variance was seen in male and female *Nnat*^+*/−p*^ mice, respectively (Fig. 4C,D).

The increase in fat mass was confirmed by analysis of fat pad mass in an independent cohort of chow fed mice. Mean gonadal fat pad mass was significantly increased in male *Nnat*^+*/−p*^ compared to wild type mice (Fig. 4E), while the difference between genotypes in female mice just failed to reach significance (*Nnat*^+*/*+^ vs *Nnat*^+*/−p*^: *P* value_females_ 0.08, Fig. 4F). Once again, in this cohort, the variance in fat pad mass between *Nnat*^+*/−p*^ and wild type was increased in both *Nnat*^+*/−p*^ sexes (Fig. 4E,F). Mean brown adipose tissue (BAT) mass from interscapular region was similar between genotypes for both sexes, but again in *Nnat*^+*/−p*^ female mice we recorded an increase of variance (Supplementary Fig. 5G,H). We concluded that the difference in body weight between genotype was driven primarily by an increased fat mass in *Nnat*^+*/−p*^ mice.

Consistent with their increased and more variable adiposity, circulating levels of leptin were higher and more variable in *Nnat*^+*/−p*^ mice when compared to wild type littermates (Supplementary Fig. 5I,J).

TD-NMR analysis performed at 11–12 weeks in a small cohort of the chow-fed male mice raised at thermoneutrality also indicated that the difference in body weight in *Nnat*^+*/−p*^ mice was driven by increased fat mass and increased variance of fat mass, while the lean mass was unchanged from *Nnat*^+*/*+^ littermates (Supplementary Fig. 2J,K).

### Transcriptomic analysis of PVN and ARC in *Nnat* deficient mice

To further explore at the molecular level how *Nnat* deficiency may contribute to the hypervariable phenotypes seen, we performed RNA sequencing from laser-capture micro dissected paraventricular nucleus (PVN) and arcuate nucleus (ARC) and compared the transcriptome between *Nnat*^+*/−p*^ mice and their *Nnat*^+*/*+^ littermates in different feeding conditions.

We performed an analysis of three subgroups: *Nnat*^+*/*+^ (all non-obese), *Nnat*^+*/−p*^ non-obese, and *Nnat*^+*/−p*^ obese mice and combined two approaches to identify differences in hypothalamic gene expression between subgroups (Supplementary Fig. 6A,B; see “Material and methods” section).

In the PVN of chow-fed males, among genes showing any difference between the three subgroups (with nominal *P* < 0.01, Likelihood Ratio Test) the largest gene expression modules (WGCNA, see “Material and methods” section) were associated with *Nnat* genotype, independently of obesity (69 genes in modules mP1, mP3; Supplementary Table 1A, Supplementary Fig. 6C).

In terms of single gene changes within the male PVN, we found 38 genes (27 up regulated, 11 down regulated) that were differentially expressed (BH-adjusted *P* value < 0.25) between obese *Nnat*^+*/−p*^ and *Nnat*^+*/*+^ mice and 2 genes (1 up, 1 down [*Nnat*]) between non-obese *Nnat*^+*/−p*^ and *Nnat*^+*/*+^ mice ( Supplementary Table 1B).

*Nnat* was among the 11 down regulated genes as expected and was found in module mP1 (Supplementary Fig. 6C). This module also included *Ntsr1*, the gene that encodes the principal receptor for the anorexigenic peptide neurotensin^23^. Genes with decreased expression in obese *Nnat*^*+/-p*^ versus *Nnat*^+/+^ mice included neurogranin, a thyroid-regulated gene whose loss has been reported to result in an anxiety-like phenotype^24^ (Supplementary Table 1B). However, module analysis of female PVN did not show a substantial overlap in constituent genes between modules identified from male and female mice (Supplementary Table 1A). In the female PVN, 133 genes (14 up, 119 down) were differentially expressed between obese *Nnat*^+/−p^ versus *Nnat*^+/+^ mice, and 193 genes (58 up, 135 down, non-obese *Nnat*^+/−p^ vs *Nnat*^+/+^ mice) respectively (Supplementary Fig. 6E and Supplementary Table 1B).

In contrast to the PVN, in the ARC of chow-fed male mice the largest gene modules were associated with obesity (62 genes in mA1, mA2) (Supplementary Fig. 6D, Supplementary Table 1A). Although our ARC *Nnat*^+*/−p*^ data set was inherently noisier than the PVN, among top-ranking genes for differential expression there was an overlap with genes differentially expressed in high fat diet males (Supplementary Fig. 7, Supplementary Table 1C-D). Of note, both *Npy* and *Tbx3* were upregulated in the ARC of obese chow fed *Nnat*^+*/−p*^ mice and obese HFD-fed *Nnat*^+*/*+^ (Supplementary Table 1D). *Npy* is a well-recognised orexigenic peptide^25^ and *Tbx3* is a gene that is expressed abundantly in hypothalamic neuronal populations, including leptin and ghrelin responsive neurons.^26^

### Membrane topology of Nnat: putative similarity of Nnat, Phospholamban, (Pln) and Sarcolipin (Sln)

We applied standard bioinformatics methods to explore the often-reported putative similarity of Nnat with the Pln and Sln. Homology searches such as Psi-Blast^27^ or HHpred^28^ failed to show any discernible phylogenic relationship between Nnat, Pln and Sln. Any standard multiple sequence alignment algorithm (e.g. Clustal Omega^29^) delivered pairwise alignment close to random. PolyPhobius signal and topology analysis^30^ predicted that Nnat is a non-secreted type III membrane protein with N-terminal hydrophobic helix spanning a membrane. The secondary structure prediction indicated a propensity to form a second C-terminal helix fully exposed to the cytoplasm. As Nnat resides in the endoplasmic reticulum^31,32^ we would predict its N-terminus must point to the lumen, with the two different splice variants differing only in the length or the linker between the two helices (Supplementary Fig. 8). Our predictions contrast with the structure of Pln^37,38^, which is known to be a type II membrane protein with its C-terminal helix spanning the sarcoplasmic reticulum membrane and its N-terminal helix exposed to the lumen. Its close paralogue Sln consists of only a single TM helix with identical topology. The inverse membrane orientation of Nnat and Pln and Sln are shown schematically in Supplementary Fig. 8.

### Effect of *Nnat* deficiency on glucose homeostasis and proinsulin processing

**Figure 5:**
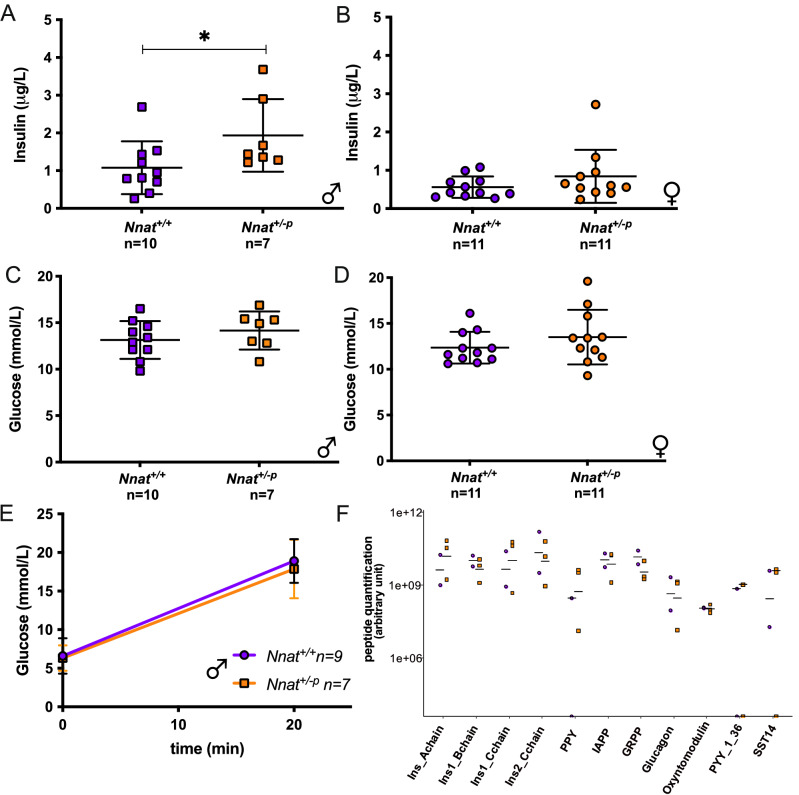
Glucose handling in *Nnat* deficiency. *Ad libitum* insulin and glucose levels in male (**A**,**C**) and female (**B**,**D**) mice fed on standard chow diet. Glucose measurements in overnight fasted *Nnat*^+*/*+^ and *Nnat*^+*/−p*^ male mice at time 0 and 20 min after glucose i.p. injection (**E**). Data are expressed as mean ± SD, *P < 0.05 for Mann–Whitney test. Mass spectrometry analysis of peptidome from *Nnat*^+*/*+^ and *Nnat*^+*/−p*^ pancreas in body weight-matched animals: (**F**) Pseudo-quantification of the main processed peptides produced in the pancreas; no major differences were detected in peptide levels. Data are expressed as mean (male; *Nnat*^+*/*+^ (n = 2), *Nnat*^+*/−p*^ (n = 3)).

As *Nnat* has been reported to be involved in glucose-stimulated insulin secretion and proinsulin processing^19^, we investigated basal and stimulated glucose and insulin levels and proinsulin processing in our *Nnat*^+*/−p*^ mice. In 13–14 weeks old mice studied in the fed state, male *Nnat*^+*/−p*^ mice had circulating insulin levels that were significantly higher than wild type littermates, while glucose levels were similar (Fig. 5A,C). No differences were detected in the female mice (Fig. 5B,D). In an independent cohort of male mice, glucose levels in the fasting state and 20 min after an intraperitoneal glucose injection were similar in *Nnat*^+*/−p*^* and Nnat*^+*/*+^ mice (Fig. 5E).

To establish whether there was any major defect in prohormone processing in the endocrine pancreas of neuronatin deficient mice, we undertook mass spectrometric peptidomic analysis, as previously described^33^ using lysates of fresh post-mortem pancreatic tissue from *Nnat*^+*/−p*^* and Nnat*^+*/*+^ mice. No systematic differences between genotypes were discernible in levels of unprocessed precursors of pre-proinsulin or other islet prohormones examined including proglucagon processed peptides, IAPP, PPY and PYY (Fig. 5F). Particular attention was given to detect insulin processing peptides and other peptides known to undergo processing from prohormones precursors to mature forms. Comparison between proinsulin and its mature form in *Nnat*^+*/*+^ and *Nnat*^+*/−p*^ tissue did not show any difference (Supplementary Fig. 9A,B). Almost all of the peptides derived from proinsulin were mature peptides. However, a peptide corresponding to the A chain peptide linked to the C-peptide was detected, indicating the presence of des 31, 32 proinsulin. This peptide was only detected in a few of the samples but showed no difference between *Nnat*^+*/*+^ and *Nnat*^+*/−p*^ tissue. As PEAKS software analysis could not identify peptides of more than 65 amino acids, we manually searched for the longer forms of proinsulin and pre-proinsulin, but we could not detect such forms. Pseudo-quantification of the main processed peptides produced in the pancreas, including proglucagon processed peptides, IAPP, PPY and PYY, did not detect any significant difference in peptide levels between genotypes (Fig. 5F). Overall, our results suggest that loss of *Nnat* does not have a major impact on the correct processing of pro-peptides produced in the pancreas.

## Discussion

Imprinted genes have long been established to play a prominent role in the disposition of energetic resources during growth and development^21^. More recently, an intriguing phenomenon has emerged from studies of body weight and mice composition in which imprinted genes have been genetically disrupted. In the case of *Nnat* and *Peg3*, genetically identical “knock out” mice display an exaggerated range of body weight and adiposity phenotypes, with some displaying normal wild type weight and others being obese. In some cases, body weights distribution has appeared to be bimodal^18^.

We confirmed previous findings that loss of the imprinted gene *Nnat* results in a bimodal body weight phenotype, with around 30% of the animals on standard chow developing obesity by 12 weeks, and also demonstrated this highly variable body weight phenotype was preserved under two other environmental conditions (45% high fat diet and thermoneutrality), driven by a high degree of variation in body fat mass.

Of note, although the increased variability was maintained on the 45% HFD, the absolute change in body weight seen over time on this diet in our study was modest and the clear bimodal pattern seen on chow was not evident on a 45% HFD. Undertaking studies with a different diet composition such as a higher fat content or a more “Western” high carbohydrate diet, may have resulted in a different pattern to that seen on 45%HFD.

At thermoneutrality, in terms of the body weight of *Nnat*^+*/−p*^ mice, both an increased variability and two clear components on clustering analysis were seen. Further, in this thermoneutrality cohort, the percentage of *Nnat*^+*/−p*^ mice considered “obese” was higher than that seen in chow. However, the small size of the cohort precluded more comprehensive analysis of the energy expenditure at 30 °C.

What was clearer was the striking hypervariability in the food intake of *Nnat*^+*/−p*^ mice in chow fed mice undergoing calorimetry studies, with a rate of weight gain in this calorimetry environment that was not representative of that seen in the usual home cage. An increase in energy intake in *Nnat*^+*/−p*^ mice was also seen in second, non-home cage system. The different effects of the novel environment on male and female mice have long been known^34^ and studies on the role of *Peg3* in maternal care and mother–pup interactions serve as an exemplar of how an imprinted gene can influence complex behaviour^35^. More recently, a fascinating complementary pattern of altered risk-taking behavioural phenotypes have emerged from studies of *Nesp55* and *Grb10*. They are both imprinted genes highly expressed at sites within the central nervous system, including the hypothalamus and monoaminergic regions of the midbrain. Mice lacking maternal *Nesp55* prefer an immediate, smaller reward and have a decreased willingness to wait for a delayed, but larger, reward^36^. The converse is seen in mice with loss of paternal *Grb10,* who are more willing to hold out for a larger, delayed reward over a smaller, immediate reward^37^. Reward processing is also altered in models of perturbed *Cdkn1c* expression, a maternally expressed imprinted gene. McNamara *et al.* reported that a transgenic model with elevated *Cdkn1* expression, mimicking loss of imprinting, showed increased motivation for palatable food stuffs and had altered expression of dopamine system-related genes in the striatum^38^. Our data raise the intriguing possibility that *Nnat* may influence feeding behaviour in a manner beyond “homeostatic” energy balance, but further behavioural studies are necessary to pursue this further.

Our transcriptomic analysis of hypothalamic regions gave some preliminary insights into the pathways which may contribute to the phenotype we observed. We saw predominantly genotype-associated expression profiles in the PVN and predominantly body weight associated profiles in the ARC. Among the PVN genes that were decreased in neuronatin deficient mice were *Ntsr1* and *UcpP2*. *Ntsr1* is the principal G-protein coupled receptor that mediates the anorectic effect of Neurotensin (Nts), a peptide that is widely expressed in the CNS with a role in several physiological processes^23^. *Ntsr1*^*−/−*^ mice have increased body weight and food intake compared to wild type mice^23^. The uncoupling protein 2 (*Ucp2)* is reported to be involved in mediating food intake regulation^39,40^ and diet-induced hypothalamic inflammation^41^. Although not addressed in our analyses, *Nnat* expression in other brain regions beyond the hypothalamus may also play a part in the regulation of food intake, appetitive behaviour and energy balance. For example, a recent study has implicated neuronatin neurons in the nucleus of the solitary tract as having a role in mediating the anorectic effect of the gut derived peptides cholecystokinin (CCK) and bombesin (BN)^42^. Further studies with selective perturbation of *Nnat* expression in different regional and neuronal population are required to build upon these observations.

Our hypothalamic gene expression analysis showed little overlap between neuronatin deficient males and females, suggesting that *Nnat* may play different roles in the two sexes. In our study, as well as some sexual dimorphic differences in the magnitude of body weight variance and postnatal growth, there was a more marked distinction in the female clusters derived from calorimetry data. *Nnat* expression had been previously recognised to have a sexually dimorphic expression pattern in the pituitary^43^ and many other imprinted genes which expressed in the hypothalamus show sexually biased or dimorphic impacts on the control of growth and body size^44^.

Our data do not directly address the molecular function of Nnat, which we believe remains to be fully resolved. However, our bioinformatic analysis fails to uncover any phylogenic homology between phospholamban/sarcolipin and Nnat. Their similarity consists solely in the fact that they are membrane embedded proteins with a transmembrane helix and a conserved phosphorylation site. Their membrane orientation is however inverse (Supplementary Fig. 8). All three proteins are reported to regulate the activity of the ion pump Serca2, a membrane embedded enzyme catalysing the hydrolysis of ATP coupled with the translocation of calcium from the cytosol to the SR or ER lumen. The actions of Pln and Sln have been well studied^45^ and depend on their phosphorylation state. Nnat also contains a phosphorylation site but its effect on Serca2 remains only putative^31,32^.

The metabolic consequences of *Nnat* deficiency have previously been reported by others, both on 129S2/Sv and C57/BL6/J backgrounds^19,20^. In contrast to our findings here, the initial report by Millership et al.^19^ reported that global loss of *Nnat* on a C57/BL6/J background had no effect on body weight up to 20 weeks and no effect on feeding behaviour in 10-week-old mice, both *ad libitum* and following fasting. Intriguingly, subsequent reports of 12-week-old *Nnat*^+*/− p*^ mice on a C57/BL6/J background^20^ revealed there was a bimodal body weight phenotype with loss of *Nnat*, but with a subpopulation of *lean* rather obese mice. In addition, further comprehensive analysis of *Nnat* deletion on a 129S2/Sv background^20^ did reveal a role for *Nnat* in growth and energy homeostasis, reporting some phenotypes similar to those of our present study. In particular, *Nnat* deficient juvenile mice (aged 3–7 weeks) had reduced body weight, adult *Nnat* deficient mice displayed a hyperphagic response to fasting and there was an obesity phenotype seen in older mice, albeit after a prolonged period of high fat diet. Taken together, we believe these findings support the concept that *Nnat* can influence food intake and body weight.

In contrast, while Millership et al. found adult *Nnat* null mice on a 129S2/Sv background had reduced energy expenditure and physical activity, our data showed loss of *Nnat* to have no effect on energy expenditure in females and to result in a small increase in metabolic rate in males. Moreover, our studies indicated that *Nnat* loss increases activity levels. The phenotypic variability recorded in calorimetry systems has been elegantly discussed by Corrigan et al.^46^ and the calorimetry system, the pre-test acclimatisation period and the time spent in the systems differ in our and the Millership et al. study. Another difference in our results from the previously published study related to the impact of neuronatin loss on glucose stimulated insulin secretion and proinsulin processing on a C57BL/6J background^19^. In our model, our limited analysis of basal and glucose-stimulated insulin secretion appeared normal, and we could not detect any abnormalities in prohormone processing in pancreatic extracts, although the increase in insulin levels in *Nnat*^+*/−p*^ male mice to maintain glucose levels may be indicative of impaired insulin sensitivity.

Our analysis of the impact of *Nnat* loss on pancreatic beta cell insulin content and secretion was not as comprehensive as that of Millership et al., we have not studied animals with tissue specific loss, and we only studied a small number of mice. There were also differences in the genetic strategies used to generate the mutant mice. Our *Nnat* mutant allele contains a lacZ cassette and lacks exons 2 and 3 of the *Nnat* gene^18^, while the mutant allele used by Millership et al. deletes only exon 1.

In conclusion, we show that loss of *Nnat* in mice results in a highly variable body weight phenotype which is retained on a high fat diet and at thermoneutrality. Further, our data show *Nnat* loss is associated with a hypervariable food intake, with the effects of this phenotype varying between genetically identical individuals.

## Material and methods

### Animals

The *Nnat* null line was rederived from original colony into a new facility where mice were housed within individually ventilated cages (IVC) (Allentown Inc.). Subsequent phenotyping occurred in another facility (West Forvie Site) with mice again housed within IVCs. Animals were housed in a controlled temperature (22 °C) facilities with a 12-h light, 12-h dark schedule (lights on 7.00–19.00) and ad libitum access to food (Safe Diets, DS-105) and water, unless otherwise stated.

*Nnat*^+*/−p*^ mice were generated as previously described, by crossing wild type mothers with heterozygous fathers to obtain *Nnat*^+*/*+^ and *Nnat*^+*/−p*^ littermates^18^. Genotyping was performed as previously described, with a mix of 3 primers (SIGMA Aldrich): common forward primer (ACACTTGGGTGGGTGAAAAAGA), wild type reverse primer (GGAGGATTTCGAAAAGCGAATC) and deletion reverse primer (CTTGGGACCACCTCATCAGAAG)^18^.

For growth curves, mice were weighed weekly. The animals studied in the body weight growth curve were generated from several different breeding cohorts in the same facility over the course of a year. Due to some operational issues, body weights at some timepoints for some animals were not recorded. All data collected are shown and no data were excluded.

#### High fat diet

A cohort of male mice was switched from standard chow to 45% HFD (D12492i, Research diet Inc.) at 4 weeks of age and kept for 3 months, with body weight monitored weekly.

#### Thermoneutrality

Different batches of male mice, always containing *Nnat*^+*/*+^ and *Nnat*^+*/−p*^ littermates, were weaned into thermoneutrality (28–30 °C) at 3–4 weeks of age and followed for 4 months with body weight recorded weekly. The thermoneutrality cabinet used (Constant climate chamber HPP750, Memmert) holds home IVC cages. The mice remained in IVC cages in groups of between 2 and 5 within the cabinet. At around 10 weeks of age the mice were singly housed in the cabinet for at least 1 week as acclimatisation for study within the indirect calorimetry system at thermoneutral conditions (MetaTrace). Body composition was undertaken on live animals by TD-NMR analysis (Minispec LF90 TD-NMR, Bunker) after the calorimetry run.

#### Chow-fed studies

Mice aged 10 weeks were singly housed for 2 weeks as acclimatisation for study within the indirect calorimetry system (MetaTrace).

The mice cohort used in chow-fed room temperature calorimetry studies were drawn from mice whose body weight had been recorded for Fig. 1 and from other mice in the same facility who had been housed in an identical way. All mice studied were singly housed for 2 weeks as acclimatisation prior to the data acquisition period in the calorimetry system. Body weights were recorded at start-finish of the single housed period to make sure the mice had a stable body weight at the time of the calorimetry run. Over this acclimatisation period the average weight change over this period of acclimatisation in the mice that underwent calorimetry was 1.2 and 3.9 g in *Nnat*^+*/*+^ *and Nnat*^+*/−p*^ male and 1.7 and 2.9 g in female *Nnat*^+*/*+^ *and Nnat*^+*/−p*^. No mice that went on to calorimetry lost more than 0.2 g in the acclimatisation period but one mouse who lost more than 16% in this acclimatisation period did not proceed to calorimetry analysis.

Body weight, food and water intake parameters were measured at the beginning, at 24 h time point and at the end of the experiment (48 h). Oxygen consumption (VO_2_), carbon dioxide production (VCO_2_), respiratory exchange ratio (VCO_2_/VO_2_) (RER), and energy expenditure were assessed by the system over the course of the run. The locomotor activity was monitored for the entire 48 h period in the MetaTrace machine and counted as the number of times an infra-red beam was broken within each 5 min interval. The beams were located on the x and y axis along the base of the cage. For food intake (FI) and energy expenditure (EE), ANCOVA^47^ was performed to assess body weight/FI or EE interactions with body weight as a covariate; genotype as a fixed factor and FI or EE as a dependent variable on SPSS version 25 (25.0 https://www.ibm.com/support/pages/downloading-ibm-spss-statistics-25 IBM). Body weight used was the average body weight taken over the time in the calorimeter. Body weight change over time in the calorimeter for the animals on standard chow was as per Fig. 2, and we observed a maximum % body weight loss of 11%. After calorimetry, male mice continued to be singly housed and females were re-group housed. Within a week of the MetaTrace analysis, body composition was determined by the TD-NMR as above. Data from all mice that underwent indirect calorimetry machine were included in analyses, except for one mouse that reported a negative food intake measure due to technical error.

#### Blood and tissue collection

At around 13–14 weeks of age, tissues and blood were collected for metabolic and biochemical profiling from a separate cohort of mice. Blood was collected by cardiac puncture under terminal anaesthesia into Microtube 1.1 mL Z-Gel (Starstedt AG & Co), centrifuged at 10,000 rpm for 10 min at 4 °C and stored at − 80 °C until use. Plasma Serum was submitted and analysed in the Core Biochemical Assay Laboratory (CBAL). Mouse insulin and leptin were measured using a Meso Scale Discovery two-plex mouse metabolic immunoassay kit according to the manufacturer’s instructions and using calibrators provided by Meso Scale Diagnostics. Blood glucose was measured using approximately 2 μl blood drops using a glucometer (AlphaTrak2; Abbot Laboratories) and glucose strips (AlphaTrak2 test 2 strips, Abbot Laboratories, Zoetis).

### Behavioural testing

Zantiks units (Zantiks Ltd) were used for the refeeding study in the non-home cage environment. The unit (size (internal, mm): 140 width, 200 length, 150 height) has a lid, a clear bottom and opaque white sides and contains 3 feeder and a shelter for a mouse to hide. Body weights were matched between genotype and both sexes were studied. The mice were acclimatised to the unit for 15 min every day during the week before the experiment. Mice were fasted overnight, and the following morning placed individually into the behavioural unit for 1 h, with ad libitum food and water. A choice of either full fat condensed milk (energy composition of 22% fat, 68% carbohydrate 9% protein, 3 kcal/g) or the light option (energy composition of 0.6% fat, 87% carbohydrate 13% protein, 3 kcal/g). Body weight, milk and water intake were recorded after the experiment (1 h). Data are presented as single or total caloric intake measured (total = full fat milk + light milk).

### Laser capture microdissection

Fresh frozen brains were collected from *Nnat*^+*/*+^ and *Nnat*^+*/−p*^ male mice between 13 and 14 weeks of age. Coronal sections of 20-μm thickness were prepared on a cryostat (Bright OTF5000 cryostat) and mounted on RNase-free membrane-coated glass slides (Superfrost Plus, Thermo Fisher Scientific). Brain sections were fixed in 95% ethanol (30 s) and rehydrated in an ethanol series (75% ethanol, 50% ethanol) prior to staining with cresyl violet (Ambion). Sections were then dehydrated in another ethanol series of 50%, 75%, 95%, and 100% ethanol and left to air dry prior to laser capture. PVN and ARC, were then dissected using a PALM Microbeam Laser Capture Microdissection System (Zeiss) on a × 5 objective as previously described^48^. Briefly, tissues were captured on AdhesiveCap Clear PCR tubes (Zeiss) and stored in 80 μl of QIAzol (QIAGEN) on dry ice prior to RNA extraction. Laser capture and subsequent cDNA library preparation were performed. RNA from laser-captured nuclei was extracted using the miRNeasy Micro RNA Extraction Kit (QIAGEN). Quality and quantity of the samples were then checked on an Agilent 2100 Bioanalyzer using Agilent RNA 600 pico and/or nano chips, with a typical sample RNA concentration in the range of 1–5 ng/μl.

### Library preparation and RNAseq

Total RNA of 500 pg from each sample was used to prepare cDNA libraries using the SMARTer Stranded Total RNA-Seq Kit v2—Pico Input Mammalian (Takara Bio). cDNA libraries were quantified on an Agilent 2100 Bioanalyzer using the high-sensitivity DNA kit (Agilent) and then submitted to CRUK-Cambridge Institute for sequencing on an Illumina Hi-Seq 4000 in 1 lane. Single end reads (SE50) with an average of 9.02 million sequenced reads per sample were obtained.

### Transcriptomic analysis of PVN and Arc

Gene-level read counts were imported for expression analysis using DESeq2 (v1.24.0)^49^ separately for male PVN, male Arc and female PVN. Three samples were removed due to very low read count. Body weight of *Nnat*^+*/−p*^ mice was dichotomised into non-obese or obese (males, non-obese < 35 g, obese > 37 g; females: non-obese < 25 g, obese > 30 g) as we observed a comparable range of body weight among wildtype chow-fed or HFD-fed mice, respectively (Supplementary Figs. 6, 7). Among mice on chow diet, exploratory clusters (modules) of correlated genes were identified using WGCNA (v1.69)^50^ using *rlog*-transformed values among genes top-ranked for any effect of sample group (*Nnat*^+*/*+^, *Nnat*^+*/−p*^ non-obese, *Nnat*^+*/−p*^ obese; DESeq2, Likelihood Ratio Test). Gene-level differential expression analysis was performed using pairwise contrasts (DESeq2, Wald test, lfcShrink; counts > 1) between chow-fed groups (*Nnat*^+*/*+^, *Nnat*^+*/−p*^ non-obese, *Nnat*^+*/−p*^ obese), between 45% high-fat diet-fed mice with different genotypes (*Nnat*^+*/−p*^ HFD-fed vs. *Nnat*^+*/*+^ HFD-fed) and between wildtype mice on different diets (*Nnat*^+*/*+^ HFD-fed vs. *Nnat*^+*/*+^ chow-fed). Gene annotation was obtained from R/Bioconductor package org.Mm.eg.db v3.8.2 (https://bioconductor.org/packages/release/data/annotation/html/org.Mm.eg.db.html). Supplementary Table 1A provides the genes used for module discovery, Pearson correlations with each module eigengene (arbitrary sign), and differential expression results (genes with nominal *P* > 0.01 in all DESeq2 results are not shown).

### Peptide extraction and analysis

Peptides from pancreas homogenates were extracted as described previously (Roberts, Larraufie et al., Diabetes 2019). Briefly, mice were culled, pancreas was extracted and two biopsies of ~ 20 mg of tissue were directly placed in 250 µl 6 M guanidine HCl solution on ice. Pancreas were then homogenised within half an hour with Lyzing MatrixD (MPbio) in a FastPrep-24 Homogeniser for 2 times 40 s at 6 m s^−1^. Proteins were precipitated by adding 4 volumes of 80% acetonitrile and after centrifugation at 12,000*g* for 5 min at 4 °C, the aqueous (lower) phase was harvested and dried using a centrifugal vacuum concentrator and stored at – 70 °C until further processing. Samples were resuspended in 250 μl of 0.1% formic acid solution and peptides purified and concentrated by solid phase extraction (Waters HLB Prime µElution) and reduced and alkylated. 10 μl of the final 120 μl was analysed on a Thermo Fisher Ultimate 3000 Nano LC system coupled to a Q Exactive Plus Orbitrap mass spectrometer as previously described (Roberts, Larraufie et al., Diabetes 2019). Peptide identification was performed using Peaks v.8.0 (BSI, Waterloo, Canada), searching against the mouse SwissProt database with fixed cysteine carbamidomethylating and variable methionine oxidation, N-terminal acetylation, and pyroglutamate and C terminal amidation modifications. Peptide quantitation and manual searches were performed using Xcalibur using *m/z* values and retention times corresponding to selected peptides. Peak areas were normalised with generic internal standards and biopsy weight. Data for each mouse is the mean of the two extracted samples. The mass spectrometry proteomics data have been deposited to the ProteomeXchange Consortium via the PRIDE [1] partner repository with the dataset identifier PXD024161 and 10.6019/PXD024161.

### Statistical analysis

All values are expressed as mean ± SD. Statistical analysis was performed by using Graph Pad Prism software (GraphPad Prism, version 9.0.2, https://www.graphpad.com/support/faq/prism-902-release-notes/) or SPSS 27 (IBM 27.0.0.0 https://www.ibm.com/support/pages/downloading-ibm-spss-statistics-27), with details of the statistical tests used provided in the figure legends. Mclust 5, an R package for model-based-clustering, was used for the clustering, classification and density analysis of the body weight phenotype as described before^22^. K-means clustering analysis of phenotypical variables: body weight, energy expenditure, respiratory exchange ratio (RER), water intake, food intake and change in body weight (dBW) was used to cluster obese and non-obese *Nnat*^+*/−p*^ mice. PEAK software (2.6 × https://www.bioinfor.com/peaks-studio/) was used for the peptidomic study.

### Ethical statement

All mouse studies were performed in accordance with UK Home Office Legislation regulated under the Animals (Scientific Procedures) Act 1986 Amendment, Regulations 2012, following ethical review by the University of Cambridge Animal Welfare and Ethical Review Body (AWERB). The reporting in the manuscript follows the recommendations in the ARRIVE guidelines.

## Supplementary Information

Supplementary Table 1.

Supplementary Figures.

## Data Availability

Raw sequencing data from this study have been deposited in the GEO database with the accession number GSE171155. The mass spectrometry proteomics data have been deposited to the ProteomeXchange Consortium via the PRIDE [1] partner repository with the data set identifier PXD024161 and 10.6019/PXD024161.
