# Supplementary material for: Murine neuronatin deficiency is associated with a hypervariable food intake and bimodal obesity
Source: Sci Rep. 2021 Sep 2;11:17571. doi: 10.1038/s41598-021-96278-8 (PMC8413370; doi:10.1038/s41598-021-96278-8)

Supplementary Figure 1.

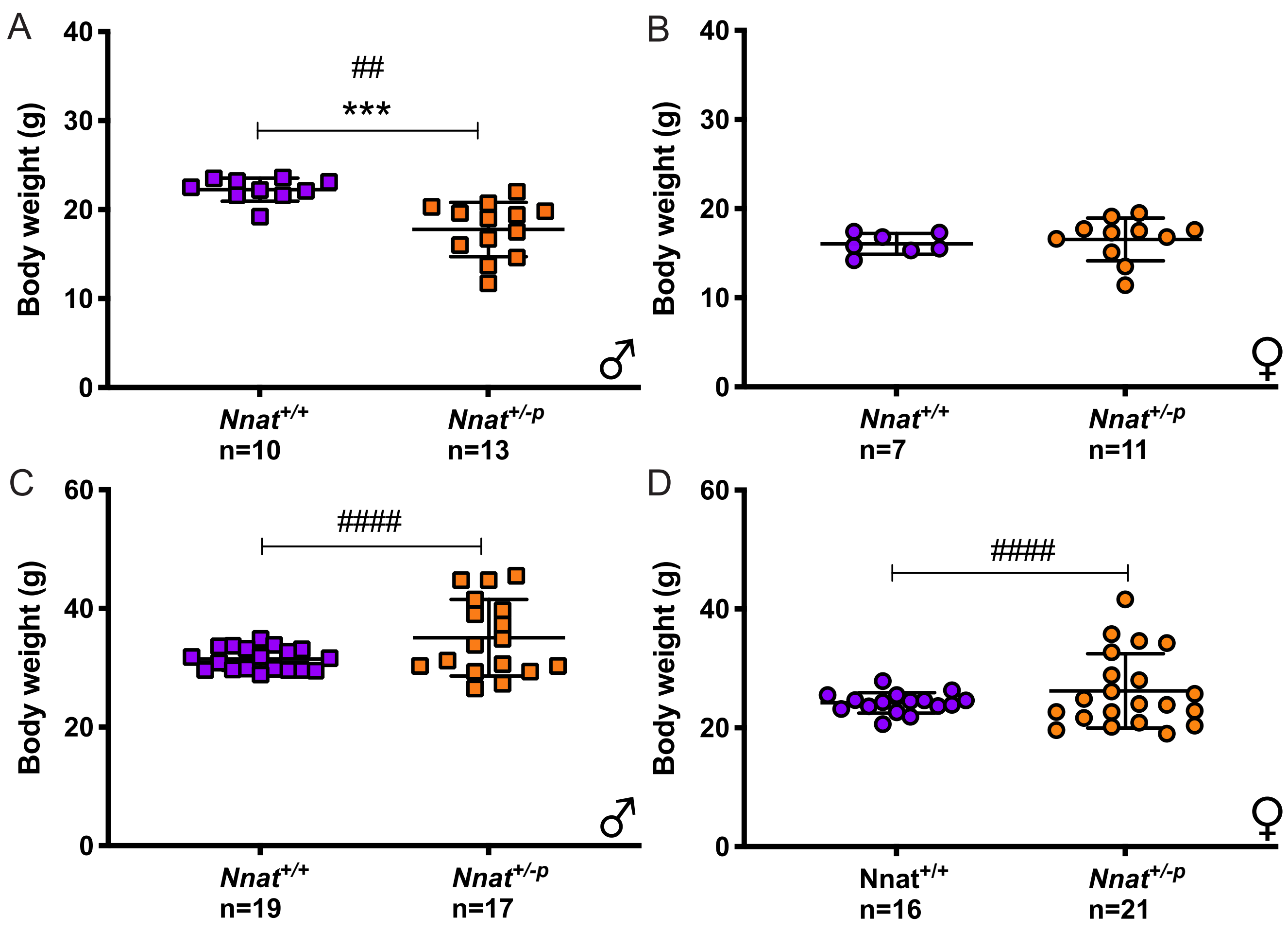

Supplementary figure 1. Body weight of *Nnat*$^{+/+}$ and *Nnat*$^{+/-p}$ male and female mice at 5 (**A, B**) and 12 (**C, D**) weeks of age fed on chow diet. Data are expressed as mean ± SD, *** *P<0.001* for Mann-Whitney test and #### *P<0.0001* for Levine's test.

Supplementary Figure 2.

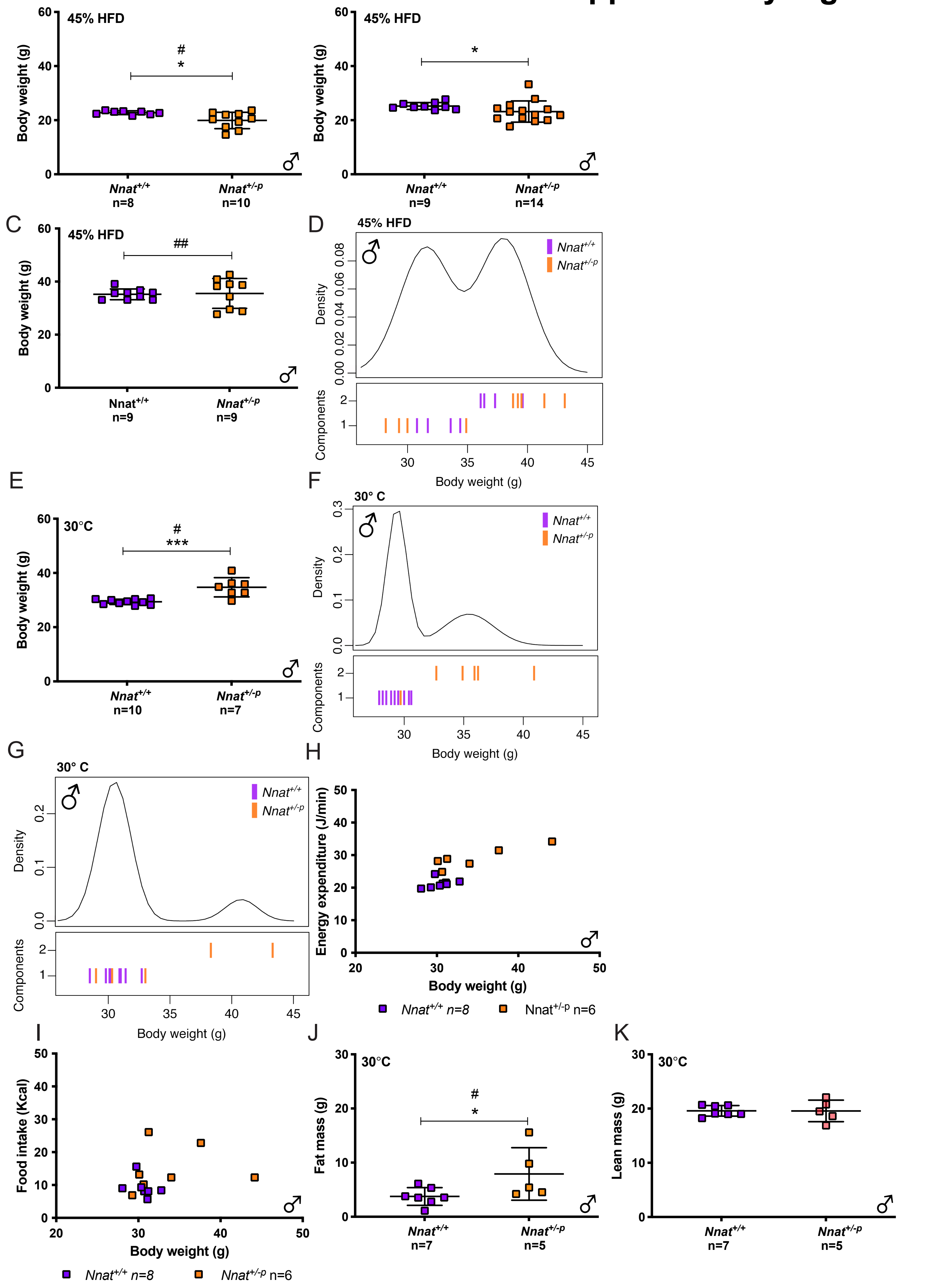

Supplementary Figure 2. Body weight of *Nnat*$^{+/+}$ and *Nnat*$^{+/-p}$ at 5 weeks (**A**) and 6 weeks (**B**) and 12 weeks of age (**C**) on 45% HFD. Mclust clustering at 12 weeks of age between *Nnat*$^{+/+}$ and *Nnat*$^{+/-p}$ male mice fed on 45% HFD (**D**) (male; *Nnat*$^{+/+}$ (n=9), *Nnat*$^{+/-p}$ (n=9)). Body weight of *Nnat*$^{+/+}$ and *Nnat*$^{+/-p}$ at thermoneutrality at 10 weeks (**E**). Mclust clustering at 10 and 12 weeks of age between *Nnat*$^{+/+}$ and *Nnat*$^{+/-p}$ male mice fed on chow diet at thermoneutrality (**F, G**) (male; *Nnat*$^{+/+}$ (n=10), *Nnat*$^{+/-p}$ (n=7)). Energy expenditure (**H**) and caloric intake (**I**) in *Nnat*$^{+/+}$ and *Nnat*$^{+/-p}$ males mice plotted against body weight (average over time in calorimetry system, mice fed on standard chow diet at thermoneutral temperature). Fat (**J**) and lean (**K**) mass recorded at 11-12 weeks of age (thermoneutrality). Data are expressed as mean ± SD, * $P<0.05$, ***$P<0.001$ for Mann-Whitney test and # $P<0.05$, ## $P<0.01$, #### $P<0.0001$ for Levine's test.

Supplementary Figure 3.

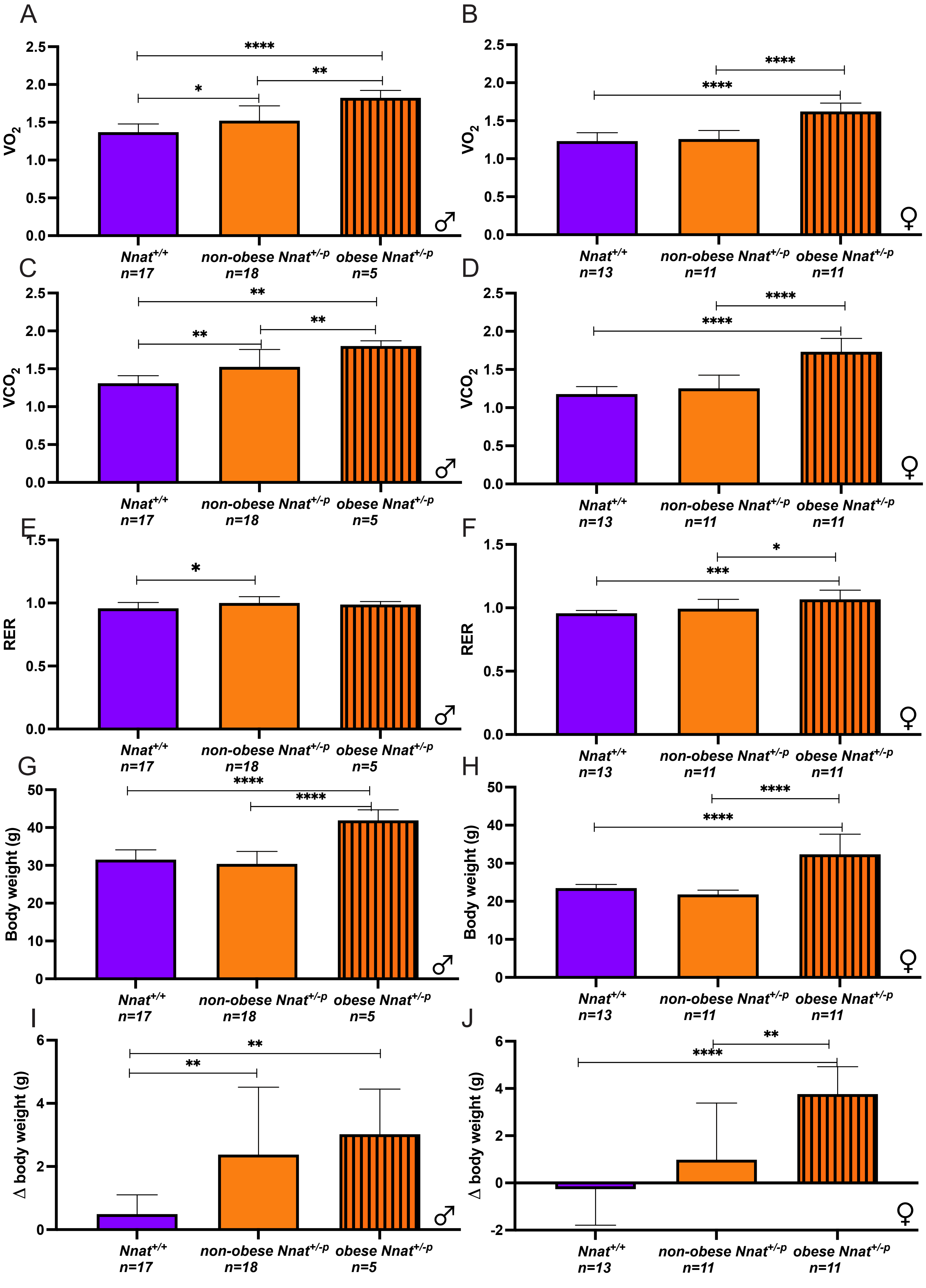

Supplementary Figure 3. (A, B) VO2, (**C, D**) VCO2, (**E, F**) RER, (**G, H**) body weight and (**I, J**) change in body weight over 48 h in calorimetry system in male and female mice fed chow standard diet. Data are expressed as mean ± SD, * $P<0.05$, ** $P<0.01$, ***$P<0.001$, ****$P<0.0001$ for Mann-Whitney test.

Supplementary Figure 4.

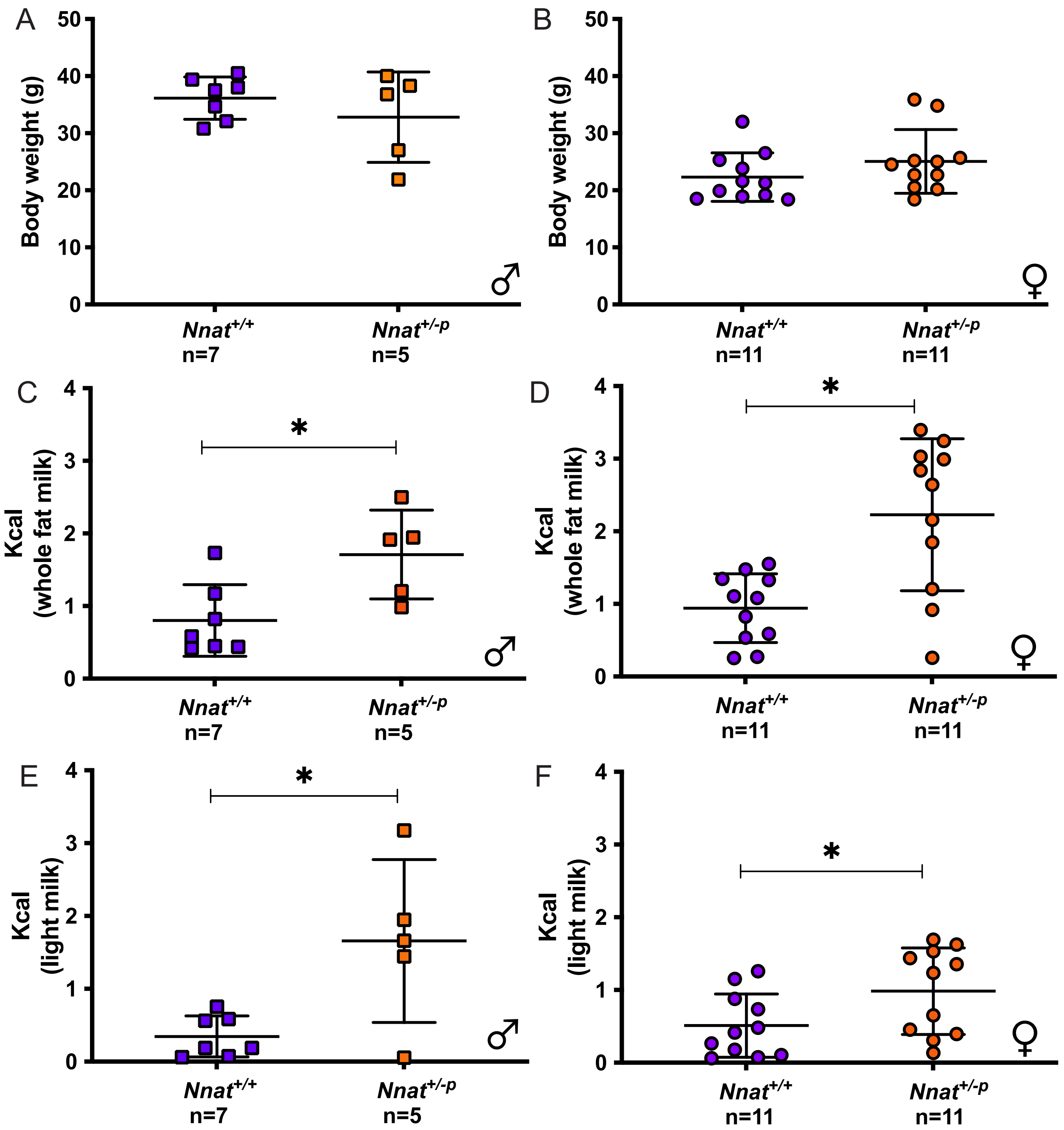

Supplementary Figure 4. Body weight of male (**A**) and female (**B**) mice studied in the re-feeding protocol in the Zantick box (condensed milk). Both *Nnat*$^{+/-p}$ males and females consume more whole fat condensed milk (**C, D**) and light milk (**E, F**) compared to *Nnat*$^{+/+}$ mice. Data are expressed as mean ± SD, **P<0.05*, ***P<0.01* for Mann-Whitney test.

Supplementary Figure 5.

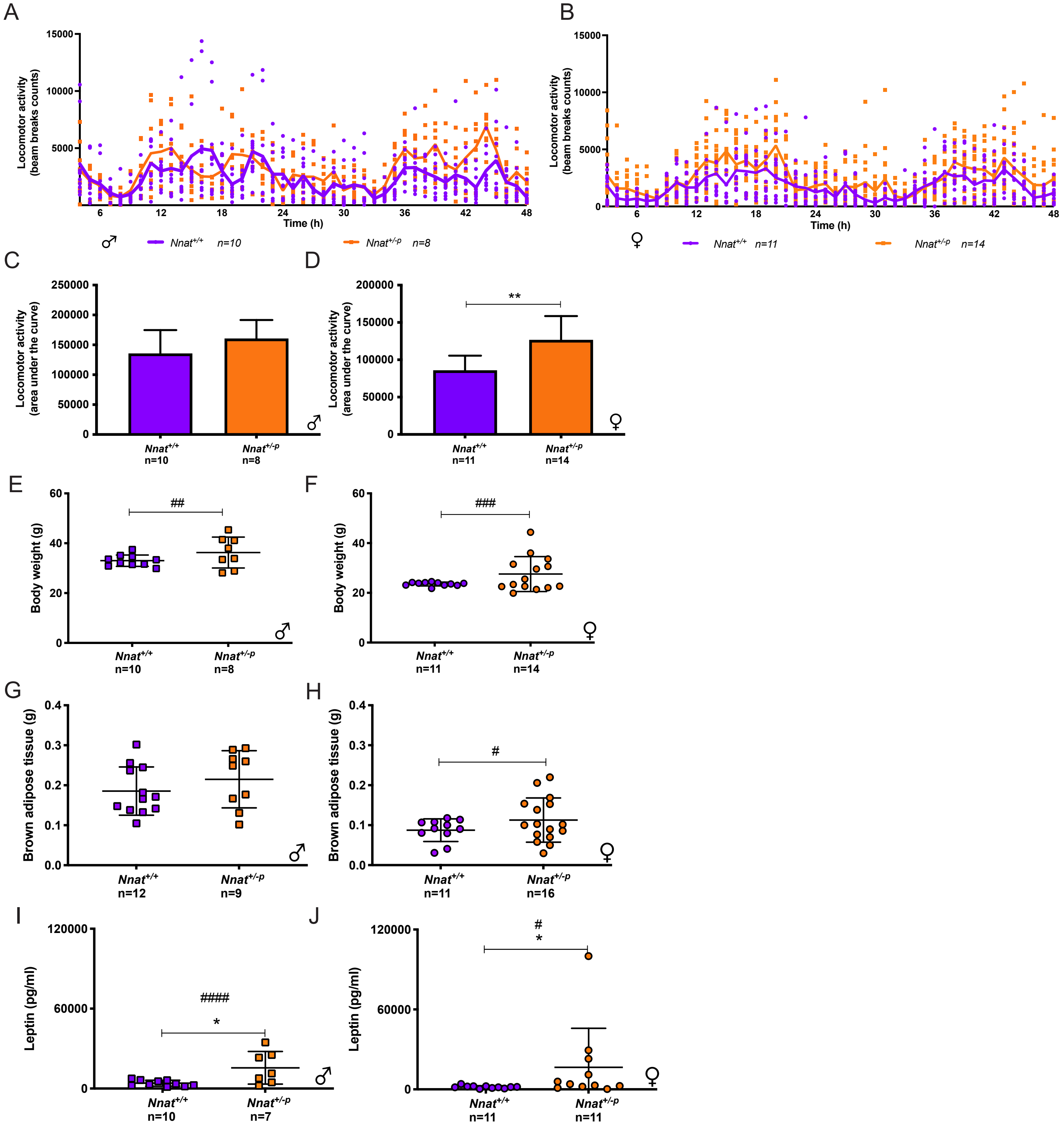

Supplementary Figure 5. *Nnat*$^{+/-p}$ mice have a higher locomotor activity compared to Nnat$^{+/+}$. Recorded locomotor activity of *Nnat*$^{+/-p}$ and *Nnat*$^{+/+}$ male (**A**) and female (**B**) mice during a period of 48 h in a calorimetry system. Analysis of the AUC of the locomotor activity in males (**C**) and females (**D**). Results are expressed as the mean hour activity in a period of 4 h ± SEM. Body weight of male (**E**) and female (**F**) mice whose activity was recorded. Weight of Brown Adipose Tissue (BAT) collected from the interscapular region in male (**G**) and female mice (**H**) at 12-13 weeks of age (chow diet, standard temperature). Leptin levels in male (**I**) and female (**J**) mice (chow diet, standard temperature). Data are expressed as mean ± SD, ** *P< 0.01* for Mann-Whitney test and ## *P<0.01, ### P<0.001* for Levine's test.

Supplementary Figure 6.

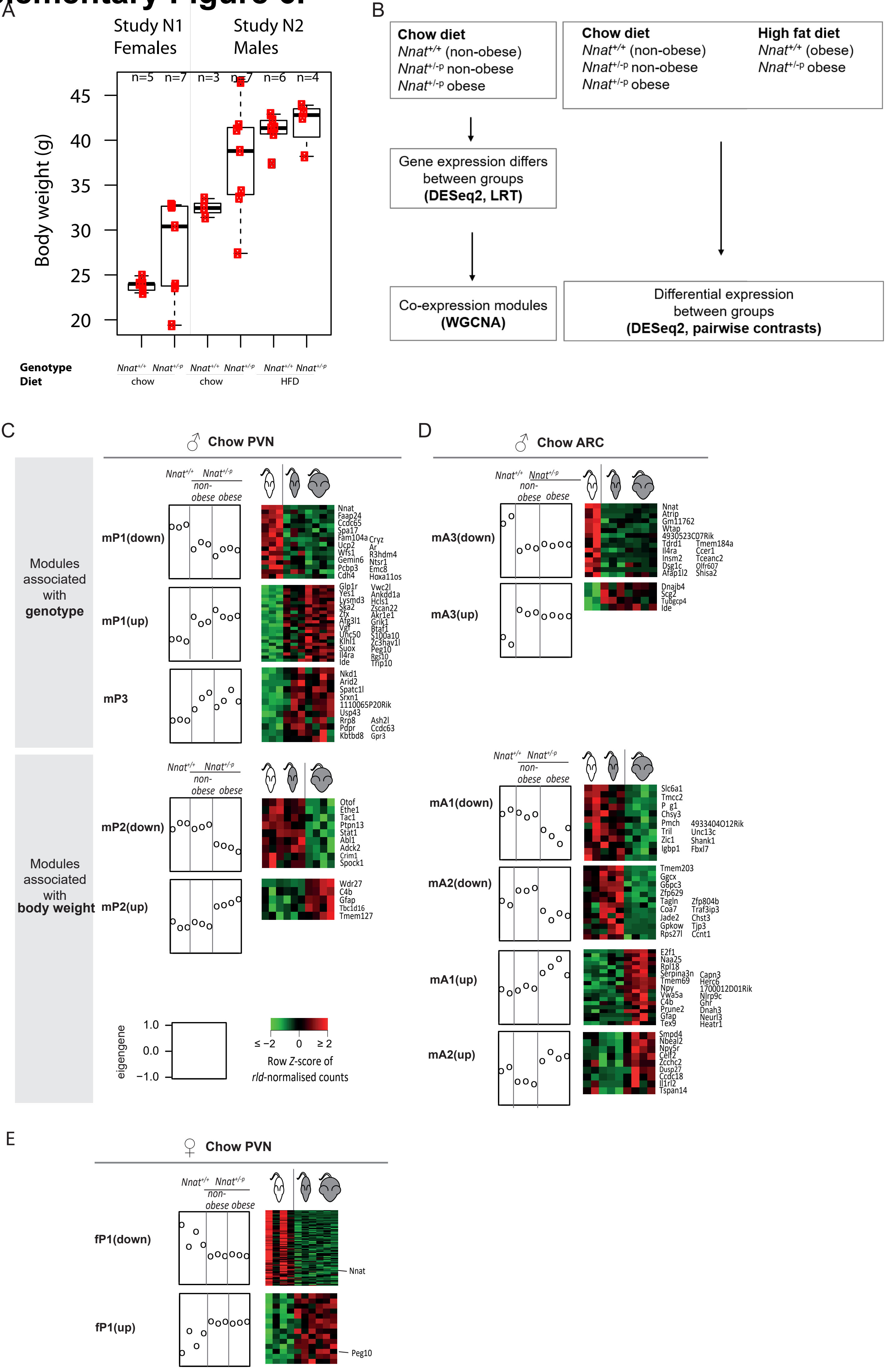

Supplementary Figure 6. Transcriptomic analysis (**A**) Body weights of female (left) and male (right) mice by genotype and diet. (**B**) Schematic showing the study design of the gene expression analysis. Full results are presented in Supplementary Table 1. (**C**) Illustration of co-expression modules from the PVN of chow-fed males which are associated with genotype (top) or body weight (bottom). Each module is split into *up* or *down* direction relative to the *Nnat*$^{+/+}$ group. Scatterplots show the module eigengene. Heatmaps illustrate the module genes which also satisfy Pearson correlation > 0.7 with the eigengene. (**D**) Illustration of co-expression modules from the arcuate nucleus (ARC) of chow-fed males which are associated with genotype (top) or body weight (bottom). (**E**) Illustration of co-expression modules from the PVN of chow-fed females which are associated with genotype.

Supplementary Figure 7.

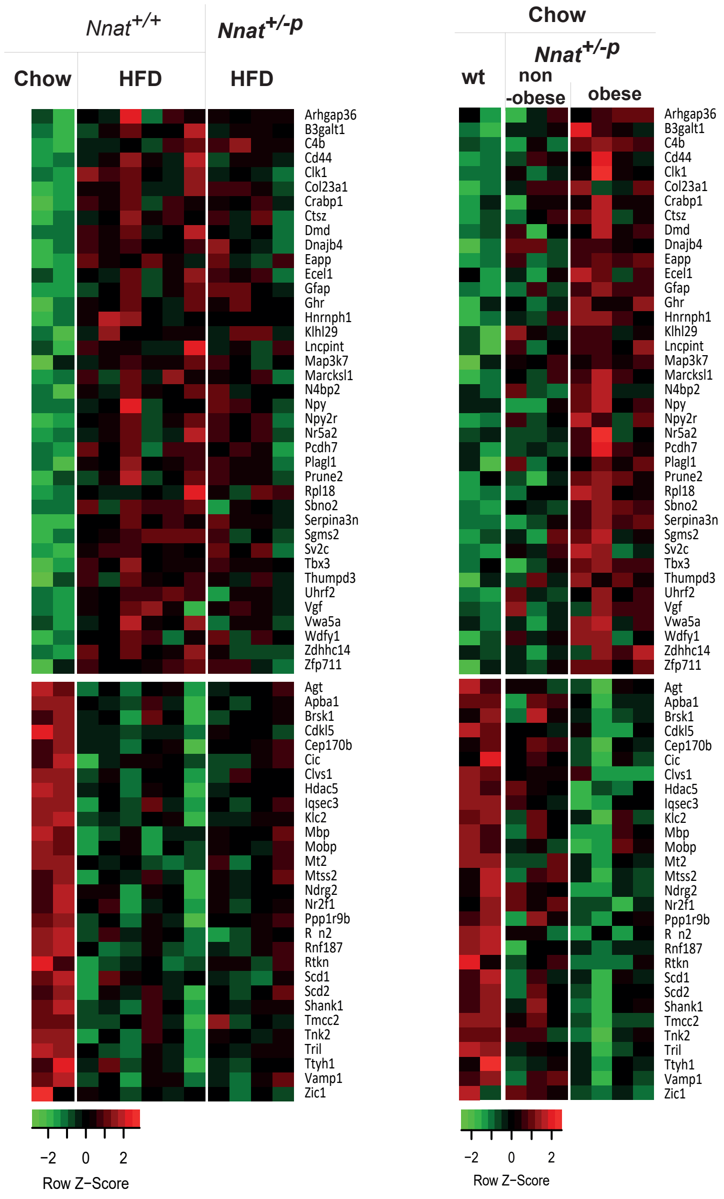

Supplementary Figure 7. In male ARC, genes differentially expressed in *Nnat*$^{+/+}$ HFD-fed versus *Nnat*$^{+/+}$ chow-fed mice (BH-adjusted $p<0.25$) and nominally differentially expressed in chow-fed obese *Nnat*$^{+/-p}$ vs *Nnat*$^{+/+}$ ($p<0.05$). Heatmaps illustrate expression profiles in chow-fed *Nnat*$^{+/+}$, HFD-fed *Nnat*$^{+/+}$, and HFD-fed Nant+/-p mice (left), and expression profiles in chow-fed *Nnat*$^{+/+}$ and *Nnat*$^{+/-p}$ mice (right). The heatmaps show rid-transformed expression values which in this visualisation are displayed as row (within-gene) Z-scores with respect to the samples displayed within each separate heatmap (left, right). Differential expression analysis is provided in Supplementary Tables 1A, 1D.

Supplementary Figure 8

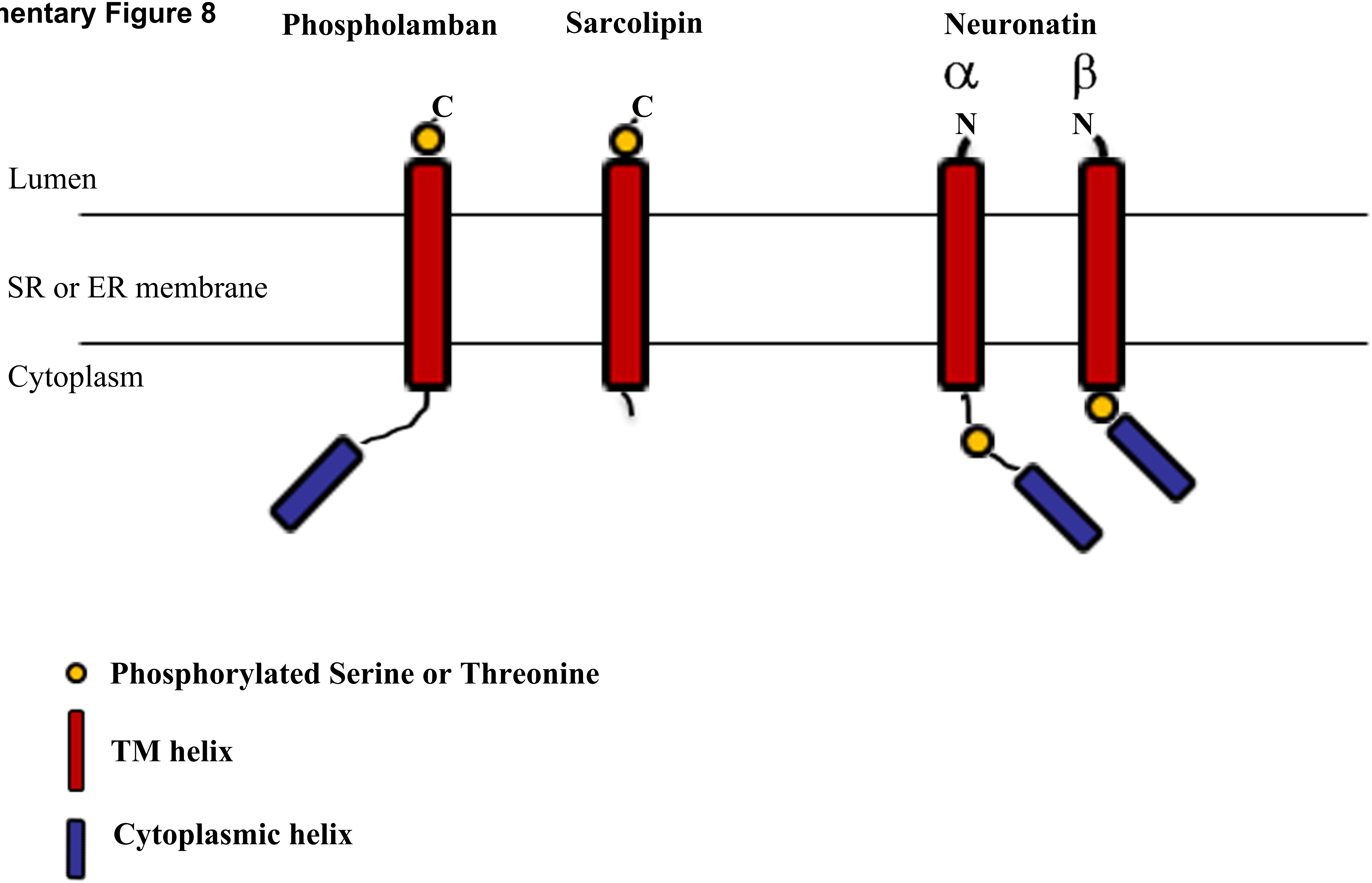

Supplementary Figure 8. Comparison of the membrane organisation of Phospholamban, Sarcolipin and Nnat (**α** and **β**) with their phosphorylation sites indicated. C, N, stand for C- and N-terminus. SR sarcoplasmic reticulum, ER endoplasmic reticulum, TM transmembrane helix.

Supplementary Figure 9.

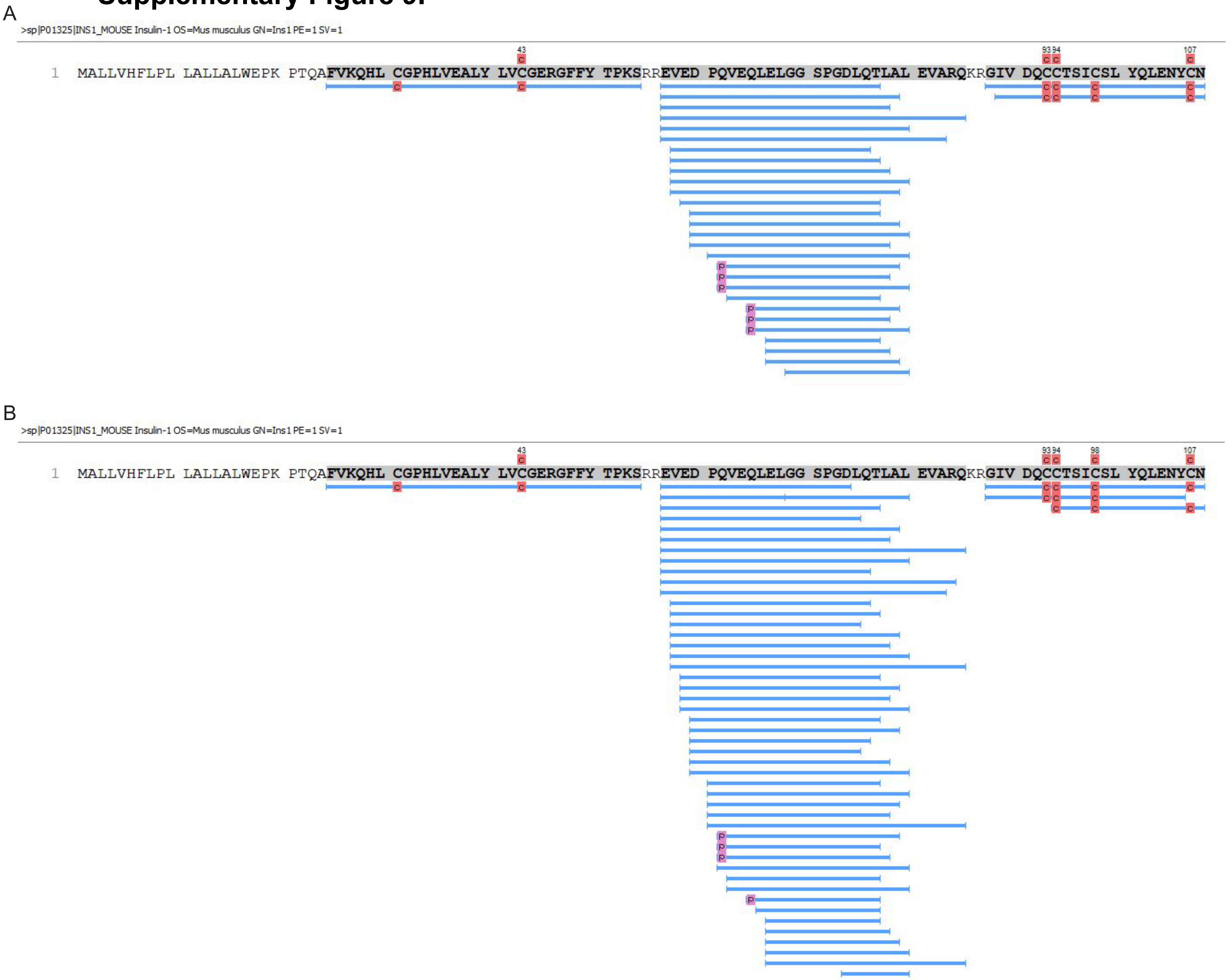

Supplement: Supplementary file 2 — Supplementary Figures. [file 41598_2021_96278_MOESM2_ESM.pdf]
